# Room for Improvement Remains in Food Consumption Patterns of Young Children Aged 2–4 Years

**DOI:** 10.1093/jn/nxx053

**Published:** 2018-06-05

**Authors:** Emily B Welker, Emma F Jacquier, Diane J Catellier, Andrea S Anater, Mary T Story

**Affiliations:** 1 Duke University, Duke Global Health Institute, Durham, NC; 2 Nestlé Research Center, Lausanne, Switzerland; 3 RTI International, Research Triangle Park, NC

**Keywords:** nutritional epidemiology, Feeding Infants and Toddlers Study, FITS 2016, dietary intake, early childhood, food intake

## Abstract

**Background:**

Healthy food consumption patterns in early childhood support optimal growth and development and promote lifelong health.

**Objective:**

The objective of the Feeding Infants and Toddlers Study (FITS) 2016 is to provide updated information on food consumption patterns of children aged 0 to <4 y. This article focuses on several key aspects of the food consumption patterns of 2- and 3-y-olds and how those patterns differ between racial/ethnic groups.

**Methods:**

The FITS 2016 is a cross-sectional study in caregivers of children aged 0 to <4 y living in the United States. Dietary data were collected in a national random sample of children (*n* = 3235, of whom 600 were aged 24–47.9 mo) by using a 24-h dietary recall telephone survey with the primary caregiver of the child. Data from the recall were used to calculate the percentage of children consuming specific food groups on the day of the recall and energy from these foods (kilocalories per consumer). Differences in food patterns between racial/ethnic groups were analyzed by using ANOVA and *t* tests.

**Results:**

On the day of the 24-h dietary recall, 27% of 2- and 3-y-olds did not consume a distinct portion of vegetables. Fried potatoes were the most commonly consumed vegetable. Approximately 75% consumed a distinct portion of fruit and 45% consumed 100% fruit juice. Eighty-one percent of children consumed cow milk. Almost all (95%) consumed a grain product, and 59% consumed a whole grain–rich product. The majority of children (88%) consumed meat or another protein food. Nearly all (90%) consumed a dessert, sugar-sweetened beverage (SSB), or sweet; and 45% consumed an SSB. Thirty-six percent of children consumed a savory snack. There were some differences in food consumption patterns between racial/ethnic groups.

**Conclusion:**

Findings from the FITS 2016 indicate that individual-, community-, and policy-level strategies are needed to improve the diets of young children in the United States.

## Introduction

Dietary habits and food preferences established early in life set the foundation for a child's lifelong eating behaviors (1–4), and often their weight and health (5–8). Infancy and early childhood represent stages of unparalleled physical, social, and emotional growth and development (9, 10). The intake of adequate food and nutrients during this period is therefore of great importance (11–15).

Yet, many young children in the United States today do not consume a diet that aligns with current dietary guidance (16–18). One indication of this misalignment is the prevalence of overweight and obesity among children in the United States; among children aged 2–5 y, almost 1 in 4 are overweight or

obese (19, 20). Although obesity prevalence has reached a plateau in this age group in recent years, wide disparities still exist: non-Hispanic (NH) black and Hispanic children aged 2–5 y are more likely to be obese than their NH white and Asian counterparts (20).

The consumption of a healthy diet consistent with the Dietary Guidelines for Americans (DGA) is a critical element in maintaining a healthy weight and preventing chronic diseases throughout all stages of life, including the early years of a child's life (14, 21). Healthy dietary patterns also support optimal physical and cognitive development in early childhood (11–15). In addition, given the relatively low energy requirements and high nutrient needs of early childhood, the consumption of energy-dense, nutrient-poor foods is particularly problematic in this age group (14, 18).

Data from the Feeding Infants and Toddler Study (FITS) 2008 showed that many 2- and 3-y-olds (i.e., children aged 24–35.9 mo and 36–47.9 mo, respectively) did not have healthy dietary patterns (18). For example, the FITS 2008 found that, during early childhood, children consumed too much saturated fat and sodium and too few nutrient-dense food groups, such as fruit, vegetables, and whole grains (18, 22). Among 2- and 3-y-olds, the most commonly consumed vegetable was French fries, and 30% of children did not consume a distinct portion of vegetables on the day of the 24-h dietary recall (18). Similarly, 27% of 2- and 3-y-olds did not consume a distinct portion of fruit (not including 100% fruit juice). Meanwhile, 86% of 2- and 3-y-olds consumed some type of sugar-sweetened beverage (SSB), dessert, or sweet or salty snack (18). It is important to explore patterns of consumption in 2016 and determine if there have been notable changes since 2008 that may warrant further investigation.

The findings from the FITS 2016 provide information on the food patterns in early childhood and may inform the development of dietary guidance in the United States. In this article, we analyze the current food and beverage intake patterns of 2- and 3-y-olds (i.e., children aged 24–35.9 mo and 36–47.9 mo, respectively), and how these food and beverage intake patterns differ among racial/ethnic groups. We also make comparisons to previously published findings from the FITS 2008 and other recent research. Such comparisons will help develop hypotheses for future trend analyses with the use of appropriate methods to examine differences in dietary patterns between the FITS 2002, 2008, and 2016. Finally, we discuss how these findings may inform targeted dietary guidance, policies, interventions, and programs to improve diets of young children.

## Methods

### Study design

The FITS 2016 is a cross-sectional study in caregivers of children aged <4 y living in the United States. This study builds on the findings and methods of FITS 2008 and 2002 (23, 24).

### Participants and recruitment

The total sample for FITS 2016 includes 3235 children from birth to age 4 y (47.9 mo). In the sample, 600 children were 24–47.9 mo old. Of these 600 children, 47% were male and 53% were female. Most of these children (65%) were NH white, 18% were NH black, 14% were Hispanic, and 2.8% were NH “other” (**Table 1**).

**TABLE 1:** Sample racial/ethnic characteristics for children aged 24–47.9 mo^1^

Age, mo	Overall, *n*	Hispanic, *n*	NH white, *n*	NH black, *n*	NH other, *n*
24–35.9	305	42	193	60	9
36–47.9	295	42	194	50	8
24–47.9	600	84	387	110	17

1 Values are numbers of observations. One respondent in each of the 2 age groups (a total of 2 in the overall age group) were missing race/ethnicity data; therefore, the columns by race/ethnicity sum to 1–2 less than the overall column. NH, non-Hispanic.

Approximately one-fourth (27%) of 2- and 3-y-olds surveyed received benefits from the Special Supplemental Nutrition Program for Women, Infants, and Children (WIC), whereas only 4.7% of their mothers received benefits from WIC. Approximately one-quarter (29%) of households surveyed with a 2- or 3-y-old received benefits from the Supplemental Nutrition Assistance Program (SNAP). Slightly more than 1 in 4 (29%) caregivers of 2- and 3-y-olds surveyed had educational levels of high school or General Equivalency Diploma or less. More than half (58%) of the 2- and 3-y-olds surveyed attended day care or preschool. This article includes results from children aged 24–47.9 mo (*n* = 600).

The recruitment phase for the FITS consisted of screening, obtaining informed consent, and conducting a recruitment interview. Data collection occurred between June 2015 and May 2016. Respondents were sampled from the following sampling frames: *1*) a targeted list from a commercial vendor (Experion, Inc.), *2*) an address-based sampling frame, *3*) a targeted cell phone frame, and *4*) a Web panel. The sampling frame is described in detail elsewhere in this supplement issue (25). A recruitment interview, which collected data about respondent and child characteristics, general feeding practices, active play, physical activity, television viewing, and sleep habits, was completed by telephone or online.

### Instruments

Instruments and methods were pilot tested over the course of 6 mo from January to May 2015. The questionnaires used were largely consistent with FITS 2008; however, some additional questions were included to reflect recent scientific findings or emerging topics of importance. The full survey instrument included 4 parts: *1*) a screener questionnaire to identify eligible respondents, *2*) a recruitment questionnaire consisting of lifestyle and sociodemographic questions, *3*) a feeding practices questionnaire, and *4*) one or two 24-h dietary recall interviews. All aspects of the instrument and recruitment materials were available in both English and Spanish. The instrument was reviewed and approved by the institutional review boards of RTI International, the University of Minnesota Nutrition Coordinating Center, and the Docking Institute of Public Affairs, Fort Hays State University.

### Procedures

Before the dietary interview, respondents were sent a packet of study materials, which included an explanatory letter, a food model booklet, a ruler, a liquid measuring cup, and instructions on how to complete information concerning foods and drinks consumed while the child was in child care. Dietary interviews consisted of a 24-h dietary recall collected by telephone with the primary caregiver, administered by certified interviewers from the University of Minnesota's Nutrition Coordinating Center by using the Nutrition Data System for Research (NDSR 2015; University of Minnesota).

A second 24-h dietary recall was collected for a random subsample of 25% of the total sampled population. Respondents for the second 24-h dietary recall were selected at random during the recruitment phase. To aid in recruitment, all respondents were offered honorariums to participate ($10 for recruitment interview, $40 for a first 24-h dietary recall, and $25 for a second 24-h dietary recall). More detailed information on the data collection procedures can be found in Anater et al. (25).

### Measures

With the use of the 24-h dietary recall data, the following outcomes were calculated: percentage of children consuming specific food groups on a given day and energy from food groups (mean kilocalories per consumer).

### Data analysis

Detailed methods of the data analysis have been previously described (25). All foods and beverages reported in the 24-h dietary recall were assigned to food groups consistent with those used for food group analysis in the FITS 2008, but were updated and expanded to incorporate new foods and beverages reported and to bring the food group classification system closer to the “What We Eat in America” food group classification used in NHANES (26). A complete list of all 340 food groups is provided in **Supplemental Table 1**.

Consistent with the approach used in the FITS 2008, mixed dishes and blended products that contain a combination of major food groups, such as soups, burritos, sandwiches, and pizza, were not classified into their constituent food groups but instead classified as “mixed dishes.” There were some exceptions when the respondent provided a recipe for the mixed dish, in which case the component foods were assigned to food groups. The estimated percentage of children consuming specific foods or food groups was calculated on the basis of a single 24-h dietary recall, which has been confirmed elsewhere as appropriate for estimation at the population level (27). SUDAAN (release 11; RTI International) software was used to account for the complex sampling design and to calculate SEs.

If the number of consumers and nonconsumers in each racial/ethnic group was ≥30, we tested the null hypothesis of no racial/ethnic difference with an ANOVA. If this hypothesis was rejected, tests for differences between any 2 subgroups were conducted with *t* tests. Adjustments were not made for multiple comparisons, so any tests of significance should be considered exploratory in nature. Survey estimates were not adjusted for comparison between surveys at this stage, because the purpose herein was to identify initial differences in food consumption patterns that may warrant further exploration.

## Results

### Milk and milk products

**Table 2** provides an overview of milk and milk-product consumption by race/ethnicity. Data by 1-y age groups are shown in **Supplemental Table 2**. Most 2- and 3-y-old children (84%) consumed some form of milk on the day of the 24-h dietary recall. The most commonly consumed type of milk on the day of the recall was 2% milk (30% consumed this type of milk). Whole milk was the second most commonly consumed type of milk (26%). Few children consumed skim milk (3.6%) or plant milks (e.g., soy milk, almond milk) and dairy substitutes (4.5%). Flavored milks were consumed by 15% of children. More than one-fourth (27%) of children consumed yogurt on the day of the recall, and 40% consumed cheese. Among children who drank milk on the day of the recall, the mean energy intake from milk was 210 kcal (Table 2). A significantly higher percentage of NH white children (86%) consumed cow milk compared with NH black children (75%). Among consumers, NH white children also consumed more energy from any cow milk and 2% milk (213 and 167 kcal, respectively) than did NH black children (172 and 117 kcal, respectively). The percentage of NH white children (6%) who consumed skim milk was significantly greater than the percentage of NH black children (0%) who did so. Significantly fewer NH black children consumed cheese and yogurt than their Hispanic and NH white counterparts.

**TABLE 2:** Consumption of milks and milk products in children aged 24–47.9 mo^1^

	Consumers,^2^ %				Energy,^3^ kcal/consumer			
Food	Overall	Hispanic	NH white	NH black	Overall	Hispanic	NH white	NH black
Any liquid milk^4^	84 ± 2.0	86	86^#^	75^#^	210 ± 9.5	205	210	175
Any cow milk^5^	81 ± 2.1	83	82^#^	71^#^	212 ± 9.7	210	213^#^	172^#^
Whole cow milk^6^	26 ± 2.4	25	26	19	238 ± 20.6	192	239	211
2%/reduced-fat cow milk^6^	30 ± 2.5	31	30	28	156 ± 10.0	158	167^#^	117^#^
1%/low-fat cow milk^6^	22 ± 2.1	26	22	24	165 ± 12.2	175	157	131
Nonfat/skim cow milk^6^	3.6 ± 0.8	2.3	6^#^	0^#^	101 ± 17.1	44^7^	112^7^	NA^7^
Plant milks/dairy substitutes^8^	4.5 ± 0.9	4.0	5.9	3.9	112 ± 20.5	41^+^^,^^	115^+^	239^^^
Any flavored milk^9^	15 ± 2.1	21^^^	14	9.1^^^	159 ± 15.0	159	182	146
Cheese	40 ± 2.6	40^^^	46^#^	22^#,^^	109 ± 10.7	120	103	105
Yogurt^10^	27 ± 2.5	37^^^	27^#^	13^#,^^	115 ± 5.4	114	120	107

1 +Hispanic different from NH white, ^#^NH white different from NH black, and ^^^Hispanic different from NH black: all *P* < 0.05. NA, not applicable; NH, non-Hispanic.

2 Values are mean percentages of children consuming the food category during a single 24-h recall (±SEs for overall).

3 Values are mean kilocalories per consumer of the food category during a single 24-h recall (±SEs for overall).

4 Includes all noninfant liquid milks, including cow milk, goat milk, and plant milks or dairy substitutes; excludes human milk (breast milk), infant formula, and toddler milk drinks, which are not consumed among this age group.

5 Includes all fat contents, as well as flavored, unflavored, or powdered.

6 Includes only unflavored cow milk of specified fat content; excludes flavored and powdered.

7 Insufficient NH black observations to conduct significance tests. Two-way test between NH white and Hispanic was not significant.

8 Includes soy milk, almond milk, and other plant-based dairy substitutes; may be flavored or unflavored.

9 Includes flavored cow milk, flavored plant milks, and flavored dairy substitutes.

10 Excludes baby-food yogurts, which are not consumed among this age group.

#### Grains

**Table 3** provides an overview of grains and grain-product consumption by race/ethnicity. Data by 1-y age groups are shown in **Supplemental Table 3**. Almost all (95%) 2- and 3-y-old children consumed some form of grain or grain product, and more than half (59%) consumed whole grains containing ≥50% of their composition as whole grain (whole grain–rich) (Table 3). On the day of the 24-h dietary recall, children who consumed grains or grain products consumed an average of 256 kcal/consumer from this food group, and consumers of whole grain–rich varieties consumed an average of 147 kcal/consumer (Table 3). Approximately half of the children consumed cereal, 41% consumed whole grain–rich cereal, and 13% consumed non–whole-grain-rich cereal. Approximately 30% consumed presweetened cereal. Approximately half of the children consumed bread, rolls, tortillas, bagels, or biscuits, with 20% consuming whole grain–rich varieties. Approximately 1 in 3 children consumed pretzels, crackers, or rice cakes (33%), with very few children (3.0%) consuming whole grain–rich varieties. Approximately one-quarter of children consumed pancakes, waffles, or French toast (23%), and very few (4.2%) consumed whole grain–rich varieties. Slightly more than one-quarter of children consumed rice or pasta (28%); however, brown rice and whole grain–rich pasta were less commonly consumed (3.5% and 1.5%, respectively). There were no significant differences in the percentage of children consuming the general categories of any grain product or any whole grain–rich product among the racial/ethnic groups analyzed. Among consumers of grains and grain products, NH white children consumed more energy (272 kcal) from any grains or grain product than did Hispanic children (218 kcal). Among consumers of cereal, NH black children consumed more energy from cereal (127 kcal) than did Hispanic children (91 kcal). The percentage of NH black children who consumed cereal (69%) was also higher than for Hispanic and NH white children (51% and 49%, respectively). A higher percentage of NH black children (40%) consumed non–whole-grain-rich cereal than did Hispanic and NH white children (24% and 28%, respectively). The percentage of NH black children who consumed whole grain–rich breads, rolls, biscuits, bagels, and tortillas was significantly lower (4.9%) than the percentage of NH white and Hispanic children who did so (21% and 23%, respectively).

**TABLE 3:** Consumption of grains in children aged 24–47.9 mo^1^

	Consumers,^2^ %				Energy,^3^ kcal/consumer			
Food	Overall	Hispanic	NH white	NH black	Overall	Hispanic	NH white	NH black
Any grains or grain product	95 ± 1.0	96	93	96	256 ± 9.4	218^+^	272^+^	241
Any whole grain–rich food^4^	59 ± 2.6	56	57	64	147 ± 8.4	136	158	136
Family cereal (RTE or hot)^5^	52 ± 2.7	51^^^	49^#^	69^#,^^	110 ± 5.2	91^^^	113	127^^^
Whole grain–rich cereal^4^	41 ± 2.7	28	23	31	114 ± 6.1	97	114	131
Non–whole-grain-rich cereal^4^	13 ± 1.6	24^^^	28^#^	40^#,^^	86 ± 7.6	69^+^^,^^	95^+^	99^^^
Presweetened cereal	29 ± 2.4	36^^^	40^#^	53^#,^^	110 ± 7.0	116	110	120
Not presweetened cereal	26 ± 2.4	16	11	19	98 ± 6.7	64^+^^,^^	104^+^	128^^^
Breads, rolls, biscuits, bagels, and tortillas	53 ± 2.7	50	57^#^	42^#^	133 ± 7.1	134	129	122
Whole grain–rich breads etc.^4^	20 ± 2.3	23^^^	21^#^	4.9^#,^^	103 ± 8.9	94	115	97
Crackers, pretzels, and rice cakes	33 ± 2.5	26^+^	42^+^^,#^	19^#^	120 ± 9.3	113	130	95
Whole grain–rich crackers etc.^4^	3.0 ± 0.7	1.7	4.7	1.9	194 ± 60.4	501	148	87
Pancakes, waffles, French toast	23 ± 2.3	29	23	23	152 ± 11.5	134	146	129
Whole grain–rich pancakes etc.^4^	4.2 ± 1.0	5.9	4.2	2.5	154 ± 36.6	107	133^#^	93^#^
Rice and pasta	28 ± 2.6	35^+^	21^+^	25	115 ± 11.7	80^+^^,^^	135^+^	122^^^
Rice	16 ± 2.3	18	10	18	107 ± 17.1	67^^^	113	119^^^
Whole grain–rich rice^4^	3.5 ± 0.9	4.6	2.9	4	78 ± 17.7	71	56	167
Pasta	8.1 ± 1.4	4.9	11^#^	2.2^#^	125 ± 18.3	103	137	147
Whole grain–rich pasta^4^	1.5 ± 0.5	1.1	2.1	1	121 ± 18.9	174^+^	115^+^^,#^	64^#^
Other-grain mixed dish	1.5 ± 0.6	1.2^6^	1.1^6^	4.7^6^	146 ± 61.7	4.0^6^	60^6^	293^6^

1 +Hispanic different from NH white, ^#^NH white different from NH black, and ^^^Hispanic different from NH black: all *P* < 0.05. NH, non-Hispanic; RTE, ready-to-eat.

2 Values are mean percentages of children consuming the food category during a single 24-h recall (±SEs for overall).

3 Values are mean kilocalories per consumer of the food category during a single 24-h recall (±SEs for overall).

4 “Whole grain–rich” includes products within a category that are ≥50% whole grain. “Non–whole-grain-rich” includes products within a category that are <50% whole grains.

5 Includes any RTE or hot cereal; excludes infant cereals, which are not consumed among this age group.

6 Insufficient observations to conduct significance tests.

#### Vegetables

**Table 4** provides an overview of vegetable consumption by race/ethnicity. Data by 1-y age groups are shown in **Supplemental Table 4**. On the day of the 24-h dietary recall, 27% of 2- and 3-y-olds did not eat a distinct vegetable portion (Table 4). Of those who did, cooked vegetables were more commonly consumed than raw vegetables. Only 15% consumed dark-green vegetables and less than one-fourth (24%) consumed orange or red vegetables. More 2-y-olds than 3-y-olds consumed dark-green or orange or red vegetables (44% compared with 34%; Supplemental Table 4). Among children who ate dark-green and orange or red vegetables on the day of the recall, the mean energy intakes were 18 and 23 kcal, respectively (Table 4). The mean daily energy intake, per consumer, of fried potatoes was 115 kcal. Nearly 1 in 3 children (32%) consumed white potatoes on the day of the recall, and 1 in 5 (19%) consumed fried potatoes. The most commonly consumed vegetable was fried potatoes; however, 4 of the 6 most commonly consumed vegetables (carrots, broccoli, tomatoes, and green beans) were nonstarchy vegetables (**Table 5**). There were no significant differences between the racial/ethnic groups analyzed in the percentage of children consuming or the energy consumed for the general category of any vegetable. There were also no significant racial/ethnic differences in the consumption of dark-green vegetables. Significantly more NH white children (29%) consumed orange or red vegetables than did NH black children (14%), but there were no differences in energy consumed. More NH black children consumed white potatoes (46%) and mashed or other potatoes (24%) than did NH white (30% and 10%, respectively) and Hispanic (28% and 10%, respectively) children.

**TABLE 4:** Consumption of vegetables in children aged 24–47.9 mo^1^

	Consumers,^2^ %				Energy,^3^ kcal/consumer			
Food	Overall	Hispanic	NH white	NH black	Overall	Hispanic	NH white	NH black
Any vegetable^4^	73 ± 2.5	72	74	80	77 ± 4.2	75	76	97
Cooked vegetables^5^	47 ± 2.7	45	46	57	37 ± 2.6	35	38	43
Raw vegetables^5^	20 ± 1.9	18	26	15	20 ± 2.0	25	19	15
Dark-green vegetables^6^	15 ± 1.9	17	13	17	18 ± 2.8	17	21	26
Orange and red vegetables^7^	24 ± 2.1	22	29^#^	14^#^	23 ± 2.5	23	24	19
White potatoes	32 ± 2.5	28^^^	30^#^	46^#,^^	109 ± 6.2	120	111	110
Baked/boiled	3.9 ± 1.2	1	3.5	4.3	67 ± 10.1	137^+^^,^^	61^+^	68^^^
Mashed/other potato mixtures	11 ± 1.4	10^^^	10^#^	24^#,^^	94 ± 10.1	108	89	90
Fried potatoes^8^	19 ± 2.2	17	18	21	115 ± 8.1	122	121	119
Other starchy vegetables^9^	12 ± 1.7	10	13	19	45 ± 5.5	25^+^^,^^	48^+^	58^^^
Other vegetables^9^	32 ± 2.4	32	33	37	23 ± 2.2	31	18	23

1 +Hispanic different from NH white, ^#^NH white different from NH black, and ^^^Hispanic different from NH black: all *P* < 0.05. NH, non-Hispanic.

2 Values are mean percentages of children consuming the food category during a single 24-h recall (±SEs for overall).

3 Values are mean kilocalories per consumer of the food category during a single 24-h recall (±SEs for overall).

4 Includes any vegetable, including white potatoes.

5 Excludes white potatoes; also excludes baby food, which is not consumed among this age group.

6 Includes broccoli, Brussels sprouts, greens, and spinach; excludes baby food, which is not consumed among this age group.

7 Includes beets, carrots, squash, sweet potato, red peppers, tomatoes, and tomato sauce; excludes baby food, which is not consumed among this age group.

8 Includes French fries and any other kind of fried potatoes.

9 Includes corn, green peas, and other starchy vegetables other than white potatoes; excludes baby food, which is not consumed among this age group.

10 Includes asparagus, cabbage, cauliflower, celery, cucumber, green beans, lettuce, green salad, mushrooms, onions, pea pods, peppers (not red), zucchini/summer squash, and vegetable mixtures; excludes baby food, which is not consumed among this age group.

**TABLE 5:** Most commonly consumed fruit and vegetables in children aged 24–47.9 mo^1^

	Consumers, %
Vegetables	
Fried potatoes^2^	19 ± 2.2
Carrots	13 ± 1.6
Broccoli	12 ± 1.8
Green beans	12 ± 1.7
Potato mixtures^3^	11 ± 1.4
Tomatoes^4^	10 ± 1.5
Fruit	
Fresh bananas	31 ± 2.7
Fresh apples^5^	28 ± 2.4
Fresh grapes	22 ± 2.4
Canned applesauce	15 ± 1.7
Fresh oranges	11 ± 1.6
Fresh strawberries	11 ± 1.6

1 Values are mean percentages ± SEs of children aged 24–47.9 mo consuming the food category during a single 24-h recall. For the 2016 study, “most commonly consumed” is defined as any fruit or vegetable reported as consumed by ≥10% of respondents; if that results in <5, the top 5 are listed. Excludes subtotals or totals such as “dark-green vegetables” or “berries.” The 2008 study reported only the top 5, regardless of percentage consuming.

2 Includes French fries and any other kind of fried potatoes.

3 Includes mashed potatoes.

4 Includes tomato sauce.

5 Excludes applesauce.

#### Fruit and 100% juice

**Table 6** provides an overview of fruit and 100% juice consumption by race/ethnicity. Data by 1-y age groups are shown in **Supplemental Table 5**. Most 2- and 3-y-olds (87%) consumed whole fruit or 100% fruit juice on the day of the 24-h dietary recall (Table 6). Approximately three-quarters of the children (77%) consumed fruit and 45% consumed 100% juice. Fresh or frozen fruit was more commonly consumed than canned or cooked fruit or dried fruit. Approximately 1 in 10 children (11%) consumed canned or cooked fruit that was sweetened or in syrup. Bananas were the most commonly consumed type of fruit (Table 5). Apple juice was the most commonly consumed variety of 100% fruit juice (21%), followed by citrus or a citrus juice blend (16%) and grape juice (6.9%) (Table 6). Among children who ate fruit or drank 100% juice on the day of the recall, they consumed an average of 115 kcal from fruit and 114 kcal from 100% juice per consumer (Table 6). There were no significant racial/ethnic differences in the percentage of children consuming or energy consumed of the overall category of any fruit or 100% juice. Among consumers of fruit, NH black children consumed less energy from any fruit (99 kcal) than did NH white (117 kcal) and Hispanic (125 kcal) children. In addition, fewer NH black children consumed fresh or frozen fruit (54%) than did NH white (72%) and Hispanic (71%) children. The percentage of Hispanic children who ate unsweetened fruit or fruit packed in juice (5%) or water was lower than that of NH white (14%) and NH black (12%) children. There were no significant racial/ethnic differences in the percentage of children consuming or energy consumed from the overall category for any 100% fruit juice.

**TABLE 6:** Consumption of fruit and 100% fruit juice in children aged 24–47.9 mo^1^

	Consumers,^2^ %				Energy,^3^ kcal/consumer			
Food	Overall	Hispanic	NH white	NH black	Overall	Hispanic	NH white	NH black
Any fruit or 100% juice^4^	87 ± 1.8	89	85	85	166 ± 6.4	167	165	161
Any fruit^5^	77 ± 2.3	77	79	69	115 ± 4.5	125^^^	117^#^	99^#,^^
Fresh or frozen fruit	70 ± 2.5	71^^^	72^#^	54^#,^^	98 ± 4.3	110	96	82
Canned or cooked fruit	23 ± 2.2	21	25	28	70 ± 5.2	81	65	73
Sweetened/packed in syrup^6^	11 ± 1.8	14	9.5	14	78 ± 8.7	87	80	71
Unsweetened/packed in juice or water^6^	11 ± 1.3	5^+^^,^^	14^+^	12^^^	54 ± 4.7	63	47	58
Dried fruit	5.1 ± 1.0	2^+^	7.3^+^	4.6	92 ± 12.7	65	104	78
Any 100% fruit juice^7^	45 ± 2.7	45	39	47	114 ± 6.7	107	114	136
Apple/apple juice blend	21 ± 2.3	23	19	17	100 ± 7.4	94	103	106
Grape juice	6.9 ± 1.6	8.7	4.7	10	107 ± 13.5	97	103	123
Citrus/citrus juice blend	16 ± 2.1	18	12^#^	25^#^	80 ± 8.8	59^^^	89	102^^^

1 +Hispanic different from NH white, ^#^NH white different from NH black, and ^^^Hispanic different from NH black: all *P* < 0.05. NH, non-Hispanic.

2 Values are mean percentages of children consuming the food category during a single 24-h recall (±SEs for overall).

3 Values are mean kilocalories per consumer of the food category during a single 24-h recall (±SEs for overall).

4 Includes fruit, 100% fruit juice, and baby-food fruit/100% juice.

5 Includes any fruit (not juice) that is not baby food, which is not consumed among this age group.

6 Sweetened and unsweetened apply only to canned or cooked fruit

7 Includes only 100% juices; excludes baby-food juice.

#### Meat and protein foods

**Table 7** provides an overview of meat and protein foods consumption by race/ethnicity. Data by 1-y age groups are shown in **Supplemental Table 6**. Most children (88%) consumed meat or another source of protein on the day of the 24-h dietary recall. Chicken or turkey was the most commonly consumed type of protein, with 41% of children consuming chicken or turkey and 17% consuming a breaded variety of chicken or turkey (data not shown). One-third (34%) of the children ate hotdogs, cold cuts, bacon, or sausage. Only 6.6% of children consumed fish or shellfish. Approximately half of the children consumed nonmeat protein sources, with egg and egg dishes (25%) and peanut butter, nuts, and seeds (23%) being the most commonly consumed varieties. Relatively few children consumed dried beans or peas (9.5%). Children who ate any meat or protein food on the day of the recall consumed an average of 237 kcal/consumer (Table 7). Among consumers of the different protein foods, children consumed the most energy from peanut butter, nuts, and seeds (161 kcal) and the least energy from beef (86 kcal) on the day of the survey. There were no significant differences in the percentage of children who consumed the general category of meat or other protein food among the racial/ethnic groups analyzed. The energy consumed from the 3 categories of any meat or other protein food, any meat, and chicken and turkey was higher among NH black children than in both NH white and Hispanic children. In addition, more NH black children ate chicken or turkey (52%) than did NH white children (38%) on the day of the survey. Energy intakes among those who consumed hotdogs, sausages, bacon, and cold cuts on the day of the survey were higher among NH black children (145 kcal) than in NH white children (103 kcal). In addition, NH black children consumed more energy from dried beans, peas, and legumes (212 kcal) than did both NH white (83 kcal) and Hispanic (43 kcal) children.

**TABLE 7:** Consumption of meats and protein foods in children aged 24–47.9 mo^1^

	Consumers,^2^ %				Energy,^3^ kcal/consumer			
Food	Overall	Hispanic	NH white	NH black	Overall	Hispanic	NH white	NH black
Any meat or other protein food^4^	88 ± 1.7	88	88	86	237 ± 9.8	216^^^	237^#^	328^#,^^
Any meat^5^	76 ± 2.4	78	76	82	175 ± 7.9	150^^^	171^#^	262^#,^^
Beef	13 ± 1.9	15	10	14	86 ± 7.3	78	98	105
Chicken or turkey	41 ± 2.6	44	38^#^	52^#^	159 ± 9.5	137^^^	157^#^	221^#,^^
Fish or shellfish	6.6 ± 1.3	7	5.7	7.8	111 ± 23.6	79	120	177
Hotdogs, sausages, bacon, cold cuts	34 ± 2.5	30	38	37	110 ± 7.1	117	103^#^	145^#^
Pork/ham	5.4 ± 1.4	5.2	6	7.6	121 ± 28.3	47^+^^,^^	122^+^	218^^^
Other protein sources^4^	48 ± 2.7	54	43	40	158 ± 10.9	134	180	166
Dried beans, peas, and legumes	9.5 ± 1.7	13	8	9.4	102 ± 18.3	43^+^^,^^	83^+^^,#^	212^#,^^
Vegetarian meat substitutes	0.8 ± 0.5	0^6^	0.5^6^	1^6^	89 ± 30.2	NA^6^	43^6^	17^6^
Eggs and egg dishes	25 ± 2.5	30	21	24	116 ± 6.3	124^^^	124^#^	95^#,^^
Peanut butter, nuts, and seeds	23 ± 2.4	18^6^	25^6^	15^6^	161 ± 17.8	162^6^	187^6^	162^6^

1 +Hispanic different from NH white, ^#^NH white different from NH black, and ^^^Hispanic different from NH black: all *P* < 0.05. NA, not applicable; NH, non-Hispanic.

2 Values are mean percentages of children consuming the food category during a single 24-h recall (±SEs for overall).

3 Values are mean kilocalories per consumer of the food category during a single 24-h recall (±SEs for overall).

4 Excludes cheese and yogurt, which are presented in Table 2.

5 In addition to the categories listed, includes lamb, goat, game, and organ meats, which are consumed by <1% of respondents in all age groups.

6 Insufficient observations to conduct significance tests.

#### Desserts, SSBs, and sweet and salty snacks

**Table 8** provides an overview of sweet and savory snack consumption by race/ethnicity. Data by 1-y age groups are shown in **Supplemental Table 7**. Almost all 2- and 3-y-olds (90%) consumed some type of dessert, SSB, or sweet snack on the day of the 24-h dietary recall. Approximately 1 in 3 children (36%) consumed savory snacks, and 17% consumed whole-grain varieties. Children who ate or drank a dessert, SSB, or sweet snack on the day of the recall consumed an average of 238 kcal/consumer from those foods (Table 8). Almost half (45%) of the children consumed an SSB, with fruit-flavored drinks being the most commonly consumed SSB (34%). Sweetened teas were consumed by 6.8% of children. More 3-y-olds than 2-y-olds consumed SSBs, with 49% and 41% consuming some variety of SSB, respectively. The mean energy intake, per consumer, of SSBs was 116 kcal on the day of the survey (Table 8). A notable percentage of children consumed sweet bakery items (e.g., brownies, cakes, pies, bars, and cookies) and candy on the day of the recall (40% and 29%, respectively). There were no significant racial/ethnic differences in the percentage of children who consumed the overall category of any sweets, SSBs, or desserts. Among consumers, Hispanic children consumed less energy from any sweets, SSBs, or desserts (193 kcal) than did NH white (270 kcal) and NH black (260 kcal) children. More NH white children ate ice cream, frozen yogurt, or pudding (16%) than did NH black children (4.2%); and more NH white children ate candy (36%) than did Hispanic (18%) children. More NH black children consumed the 2 categories of any SSBs and fruit-flavored drinks (62% and 55%, respectively) than did NH white (45% and 33%, respectively) and Hispanic (45% and 35%, respectively) children. In addition, among consumers of SSBs, NH black children consumed more energy from any SSBs and fruit-flavored drinks (152 kcal and 149 kcal, respectively) than did NH white (105 and 97 kcal, respectively) and Hispanic (109 and 103 kcal, respectively) children. There were no significant differences in either the percentage of children who consumed or the energy consumed from savory snacks or whole grain–rich savory snacks.

**TABLE 8:** Consumption of desserts, SSBs, and sweet or savory snacks in children aged 24–47.9 mo^1^

	Consumers,^2^ %				Energy,^3^ kcal/consumer			
Food	Overall	Hispanic	NH white	NH black	Overall	Hispanic	NH white	NH black
Any sweets, SSBs, or desserts	90 ± 1.5	91	91	90	238 ± 9.9	193^+^^,^^	270^+^	260^^^
Cereal/nutrition bars	8.0 ± 1.2	4.9^+^	11^+^	6.6	132 ± 9.7	108^+^	138^+^	152
Sweet bakery^4^	40 ± 2.6	37	45	45	173 ± 8.6	161	181	176
Ice cream, frozen yogurt, pudding	12 ± 1.5	10	16^#^	4.2^#^	181 ± 19.5	130	200	143
Gelatins, ices, sorbets	6.7 ± 1.2	5.7	7.9	6.8	59 ± 5.8	46	63	66
Candy	29 ± 2.4	18^+^	36^+^	26	103 ± 6.6	108	99	84
Milk flavorings	4.7 ± 1.1	5.8	5.6	3	29 ± 7.6	17	35	38
Sugar, syrup, preserves, jelly	36 ± 2.7	33	38	30	53 ± 5.4	64	55	40
Any sweetened beverage	45 ± 2.7	45^^^	45^#^	62^#,^^	116 ± 6.4	109^^^	105^#^	152^#,^^
Carbonated sodas	8.8 ± 1.5	10	9.3	8.2	85 ± 17.4	65	106	78
Fruit-flavored drinks	34 ± 2.5	35^^^	33^#^	55^#,^^	112 ± 6.7	103^^^	97^#^	149^#,^^
Sweetened teas	6.9 ± 1.3	6.8	8.3	7.8	41 ± 8.7	46	35	60
Other	4.0 ± 1.2	6.5	2.9^#^	0.6^#^	93 ± 16.8	59	106^#^	63^#^
Sports drinks	1.8 ± 0.7	4.7	1.1	0.6	62 ± 20.8	42^+^	105^+^^,#^	63^#^
Any savory snacks^5^	36 ± 2.6	27	37	42	128 ± 8.7	125	129	152
Whole grain-rich savory snacks^6^	17 ± 2.0	12	19	17	102 ± 9.6	96	98	110

1 +Hispanic different from NH white, ^#^NH white different from NH black, and ^^^Hispanic different from NH black: all *P* < 0.05. NH, non-Hispanic; SSB, sugar-sweetened beverage.

2 Values are mean percentages of children consuming the food category during a single 24-h recall (±SEs for overall).

3 Values are mean kilocalories per consumer of the food category during a single 24-h recall (±SEs for overall).

4 Includes cakes, pies, chocolate/sweet cookies, bars, brownies, sweet rolls, doughnuts, muffins, and quick breads.

5 Includes chips, corn chips, popcorn, snack mix, and puffs (non-baby food), regardless of whole-grain content.

6 Includes savory snacks that are ≥50% whole grain.

#### Mixed dishes

**Table 9** provides an overview of milk and milk-product consumption by race/ethnicity. Data by 1-y age groups are shown in **Supplemental Table 8**. Approximately two-thirds of children consumed some variety of mixed dishes on the day of the 24-h dietary recall (Table 9). The most commonly consumed varieties were sandwiches (18%), pizza (14%), soups (14%), macaroni and cheese (12%), and spaghetti, ravioli, and lasagna (11%).

**TABLE 9:** Consumption of mixed dishes in children aged 24–47.9 mo^1^

	Consumers,^2^ %				Energy,^3^ kcal/consumer			
Food	Overall	Hispanic	NH white	NH black	Overall	Hispanic	NH white	NH black
Any mixed dishes^4^	67 ± 2.5	72	65	66	293 ± 16.3	315	263	322
Beans and rice, other bean mixtures	4.9 ± 1.7	14	1.0	3.1	132 ± 17.4	134	132	123
Beef with vegetables and/or rice/pasta	5.0 ± 1.5	4.1	3.8	4.2	181 ± 14.1	135	179	158
Chicken or turkey with vegetables and/or rice/pasta	2.6 ± 0.7	2.1	2.9	1.3	184 ± 22.4	114	190	291
Pork/ham with vegetables and/or rice/pasta	0.4 ± 0.3	1.2	0.1	0	89 ± 11.7	80	147	NA
Fish or shellfish with vegetables and/or rice/pasta	1.7 ± 1.1	0.0	0.8	1.1	241 ± 22.7	NA	219	155
Macaroni and cheese	12 ± 1.6	12	14	10	233 ± 20.6	282	211	306
Spaghetti, ravioli, lasagna	11 ± 1.7	9.5	12	15	225 ± 20.2	247	214	254
Pizza	14 ± 1.8	18	14	8.5	288 ± 24.8	338	243	337
Burrito, taco, enchilada	7.1 ± 1.5	7.7	7.9^#^	2.0^#^	263 ± 30.9	311	234	615
Soup	14 ± 2.3	19	8.1	7.4	107 ± 9.2	91	102	156
Sandwich	18 ± 2.1	16	17^#^	28^#^	243 ± 15.2	246	227	254

1 +Hispanic different from NH white, ^#^NH white different from NH black, and ^^^Hispanic different from NH black: all *P* < 0.05. NA, not applicable; NH, non-Hispanic.

2 Values are mean percentages of children consuming the food category during a single 24-h recall (±SEs for overall).

3 Values are mean kilocalories per consumer of the food category during a single 24-h recall (±SEs for overall).

4 Excludes mixed dishes that are predominantly grains, which are presented in Table 3.

For both the percentage of children who consumed different foods and beverages and the mean kilocalories per consumer, the *P* values were attenuated in most instances upon adjusting for income. This suggests that income was an important determinant of food consumption patterns and may have accounted for some of the differences observed between race/ethnicity groups.

## Discussion

### Key findings

The 2015 DGA recommend that children aged ≥2 y consume low-fat dairy; grains, half of which should be whole grains; a variety of vegetables from all subgroups; fruit or 100% fruit juices, with an emphasis on whole fruit; and a variety of protein foods including seafood, lean meats, eggs, legumes, soy products, nuts, and seeds (14). Findings from the FITS 2016 suggest that the food consumption patterns of young children are meeting some, but not all, of these recommendations. The analysis of dietary patterns among 3 racial/ethnic groups highlighted some differences in energy consumed and percentage of children who consumed food groups and categories that could inform future targeted intervention efforts. These data should, however, be interpreted with caution, because the *P* values were not corrected for type 1 error.

Most 2- and 3-y-olds consume milk; however, there is significant room for improvement in the varieties of milk consumed. Reduced-fat (2%) milk remains the most commonly consumed variety, whereas skim milk is the least commonly consumed variety. These data are generally consistent with findings from the FITS 2008; however, even fewer children consumed skim milk in 2016 than in 2008. There were several notable differences in milk consumption by racial/ethnic group. Fewer NH black children consumed any milk and skim milk than did NH white children. Efforts are needed among all children to improve the varieties of milk consumed; however, tailored and targeted interventions may be needed specifically among NH black children and caregivers to increase low-fat milk consumption (28, 29). For example, recent research has shown disparities in price and access to low-fat milk in predominantly Hispanic and NH black communities, so food retail policies and programs, in addition to other strategies, may play an important role in shifting consumption (30). Whole and 2% milk can contribute a notable amount of saturated fat to the diets of young children, so increasing consumption of low-fat varieties will be important, particularly among children who may be at increased risk of obesity or cardiovascular disease due to family history (31, 32). Approximately 15% of 2- and 3-y-olds consumed flavored milks on the day of the 24-h dietary recall. Parents and caregivers of young children should be encouraged to offer only low-fat or non-fat varieties of milk without added sugars or noncaloric sweeteners.

Almost all children consumed grains on the day of the 24-h dietary recall (95%) and more than half (59%) consumed a whole grain–rich variety. This represents a slight decline from FITS 2008, when 97.7% of children consumed grains on the day of the survey. There were no significant differences in overall whole-grain consumption patterns among the racial/ethnic groups analyzed, suggesting that improvements are needed in whole-grain consumption among all young children. The US DGA suggest that children aged 1–3 y consume too few whole grains and too many refined grains (14). Whole grains were more commonly consumed in the forms of cereals, breads, rolls, biscuits, bagels, and tortillas and much less commonly consumed as rice, pasta, pancakes, waffles, French toast, crackers, pretzels, and rice cakes. Efforts should be made to encourage parents and caregivers of all children to choose whole-grain varieties of these foods.

Of concern, the FITS 2016 found that 27% of 2- and 3-y-old children did not consume a distinct vegetable portion on the day of the 24-h dietary recall. Similar levels of vegetable consumption were found in the FITS 2008, which reported that 30% of 2- and 3-y-olds did not consume a distinct vegetable on the day of the recall (18). The consumption of nutrient-dense varieties of vegetables such as dark-green and orange or red vegetables remains low, with only 15% and 24% of children in the FITS 2016 consuming these varieties on the day of the survey, respectively. Four of the top 6 most commonly consumed vegetables are nonstarchy vegetables (Table 5). The percentage who consumed starchy and other vegetables decreased slightly between 2008 and 2016. However, the percentage who consumed white potatoes and fried potatoes increased slightly. This indicates that there is still ample room for improvement in the consumption of both overall vegetables and the recommended varieties of vegetables. Young children should consume 1–1.5 cups of vegetables/d and 0.5–1 cups of dark-green and 2.5–3 cups of orange or red vegetables are recommended per week (14). Few differences were observed in energy consumed or the percentage of children who consumed vegetables among the racial/ethnic groups included in analyses, suggesting that interventions to increase vegetable consumption are needed among all children. More NH black children consumed white potatoes than did their NH white and Hispanic counterparts. Other studies have found similar trends of higher consumption of white potatoes and French-fried potatoes among NH black children aged 1–3 y, so tailored programs and interventions focused on reducing white potato consumption and increasing other varieties may be needed in this subpopulation (33). Further research should examine trends in vegetable consumption over time and among particular subpopulations.

The values presented here reflect only vegetables eaten as distinct food items and do not include the percentage of children who consumed vegetables as part of mixed dishes. Recent research suggests that, among 2- and 3-y-olds, mixed dishes contribute up to one-quarter of the amount of vegetables consumed, so the values presented here may underestimate actual consumption (31).

These findings on vegetable intake are consistent with other research in this age group. Data from NHANES 2007–2010 (26) found that children aged 1–3 y consumed, on average, only 0.7 cup-equivalents of vegetables/d and only 10% of boys and 15% of girls ate the recommended 1–1.5 cups of vegetables/d (14, 16). NHANES also found that children were consuming too few dark-green and orange or red vegetables, with white potatoes being the most commonly consumed vegetable variety (14, 16). Estimates with the use of NHANES 2009–2012 data suggest that children aged 1–3 y are consuming even fewer vegetables than in 2010, 0.58 cup-equivalents/d (33).

As would be expected, a higher percentage of children consumed fruit than vegetables on the day of the 24-h dietary recall, with only 13% not consuming any fruit or 100% juice. Notably, fewer children consumed 100% fruit juice (14% fewer) and more consumed whole fruit (4.7% more) than reported in the FITS 2008. Approximately 1 in 10 children consumed sugar-sweetened varieties of canned or cooked fruit. Considering that the DGA recommend limiting added sugar to 10% of calories and that children ages 2–5 y consume an average of 11.1 teaspoons of added sugar/d (14, 34), efforts should be made to encourage parents and caregivers to provide only unsweetened varieties or varieties packed in 100% fruit juice. Few differences between the racial/ethnic groups analyzed emerged in fruit and 100% fruit juice consumption. The NHANES 2007–2010 showed similar results, with the majority of children aged 1–3 y meeting the recommendations for fruit and 100% juice intake; more than half of the amount consumed was in the form of whole fruit (14, 16).

Most 2- and 3-y-olds consumed meat or another source of protein on the day of the 24-h dietary recall. One-third of the children consumed processed meats such as hotdogs, cold cuts, bacon, or sausage, which are often high in sodium and saturated fats. There were no significant racial/ethnic differences in the percentage of children who consumed the category of any meat or protein food; however, among consumers, NH black children consumed significantly more energy from this category than NH white and Hispanic children. NH black children also had higher energy intakes, among consumers, of several varieties of protein such as chicken and turkey, hotdogs, sausages, bacon, cold cuts, and beans and legumes than other racial/ethnic groups analyzed. Research suggests that most young children in the United States are meeting or exceeding the recommended intake of protein (14, 16). These data suggest that NH black children may be overconsuming specific subgroups of protein foods; however, other research that examined mean daily intake of protein in 1- to 3-y-olds found no significant racial/ethnic differences, so further research is needed (33). Preferred protein sources, such as seafood, nuts, seeds, and soy products, are not consumed by a large percentage of children (14, 16). The American Academy of Pediatrics and the 2015 DGA recommend that both seafood and nuts, seeds, or soy products be consumed ≥1 time/wk. These data suggest that what is needed is not necessarily an increase in protein consumption but rather a shift to more lean meats and seafood, nuts, seeds, soy, and legumes, which have less saturated fat. Previous research has shown that nearly 100% of children aged 1–3 y exceed the recommended limit for solid fats (16).

Young children's consumption of desserts, SSBs, and sweet and salty snacks remains a significant cause for concern. Nearly half of all 2- and 3-y-olds consumed SSBs on the day of the 24-h dietary recall. These patterns are similar to what was found in FITS 2008. Importantly, the 2016 data suggest that both the percentage of children who consume SSBs and the grams of SSBs consumed are higher in 3-y-olds (49%; mean of 310 g) than in 2-y-olds (41%; mean of 284 g). Fruit-flavored drinks were the most common variety of SSB consumed, which is consistent with findings of the FITS 2008. In addition, a higher percentage of NH black children consumed both SSBs and fruit-flavored drinks than did NH white and Hispanic children, and NH black children consumed more energy from these beverages. These trends suggest that, although programs and policies to reduce SSB consumption are needed among all young children, it may be important to target interventions specifically to NH black children and families because similar trends are seen in school-aged children (28). Young children have an innate preference for sweet and a dislike for bitter tastes; however, these preferences are malleable (35). Therefore, it is important to provide healthy beverage options such as water and low-fat milk without added sugars to promote lifelong healthy habits. In addition, research suggests that fruit-flavored drinks often contain nutrient or health claims that can be misleading to parents and caregivers (36). It will be important to educate parents and caregivers about healthy beverages and potentially require products with nutrition-related claims to meet minimum nutrition standards to help parents and caregivers make healthy choices (36). Research has also shown that advertisements for SSBs are significantly higher in areas with a high proportion of black children and low-income children, suggesting that future interventions may need to consider the impact of targeted marketing (37).

The consumption of desserts and savory snacks also remains high. There were no racial/ethnic differences in the percentage of children who consumed the overall categories of any SSB, sweet, or dessert or any savory snacks. Hispanic children consumed less energy from any sweet, SSB, or dessert than did NH black and NH white children. Dessert and snack consumption is particularly problematic in young children because discretionary calories are limited to ∼100–150 kcal/d (14). In this article, we did not analyze eating occasions and whether these foods are consumed between meals; however, previous FITS showed that 25–28% of total energy intake in early childhood comes from snacks (38). In addition, other studies in children from 2 to 18 y have shown that both the prevalence of snacking and the amount of energy consumed from snacks have increased considerably over time (39, 40). Further exploration of energy and nutrient intakes by eating occasion in early childhood is warranted.

### Improving the diets of young children

Overall, there remains significant opportunity to improve the diets of young children in the United States. Data from the FITS 2016 suggest that children are not fully meeting the recommendations set by the DGA. The implications of these findings are of considerable public health importance. Individual-, community-, and policy-level shifts are needed to improve children's diets. Examples of strategies may include exposing children to a variety of healthful foods at a young age, in addition to repeated exposure to those foods (41–44); role modeling of healthy eating by parents and caregivers (10, 45, 46); improving the food environment in child care settings (47–49); incentivizing the purchase of fruit and vegetables within SNAP; and continuing to review and improve the WIC food package to ensure that it meets the nutritional needs of women, infants, and young children (44).

### Strengths and limitations

There are many strengths of the FITS 2016. It is one of the largest studies on the feeding behaviors of young children and is based on a rigorous survey design and state-of-the-art 24-h dietary recall methods. There are also several limitations to the data presented here. These findings are based on 24-h dietary recall data reported by the caregiver of the child, who recounted all food and beverage intake during the 24-h period; some degree of reporting bias and measurement error is inherent with these methods of dietary recall.

### Conclusions

Findings from the FITS 2016 suggest that most 2- and 3-y-olds in the United States consume milk or milk products, grains, and protein foods on a given day. However, too few children consume fruit, vegetables, and whole grains, and too many children consume sweets, SSBs, and sweet and savory snacks. Given the significant impact of dietary intake in the early years of a child's life on his or her future diet, weight, and health, these findings suggest that major shifts are needed at the individual, community, and policy level to ensure that all young children have the opportunity to achieve a healthy diet and lifestyle.

## Supplementary Material

Supplement Tables

## Notes

Published in a supplement to *The Journal of Nutrition*. Select contents of this supplement were presented at the Experimental Biology 2017 conference in a session titled, “Informing B-24 Dietary Guidelines: Findings from the New Feeding Infants and Toddlers Study 2016,” held in Chicago, IL, April 22, 2017. Supplement Coordinators were William Dietz, Milken School of Public Health at the George Washington University and Andrea S. Anater, RTI International. The FITS research was funded by the Nestle Research Centre (Lausanne, Switzerland) through a contract with RTI International and its subcontractor, the University of Minnesota. Dr. Dietz is a compensated consultant to RTI on this project. The article contents are the responsibility of the authors and do not necessarily represent the opinions or recommendations of Nestle. Publication costs for this supplement were defrayed in part by the payment of page charges. This publication must therefore be hereby marked "advertisement" in accordance with 18 USC section 1734 solely to indicate this fact. The opinions expressed in this publication are those of the authors and are not attributable to the sponsors or the publisher, Editor, or Editorial Board of *The Journal of Nutrition*.

Supported by Nestlé Research Center, Lausanne, Switzerland.

Author disclosures: EBW, DJC, ASA, and MTS, no conflicts of interest. EFJ is an employee of Nestlé Research Center.

Supplemental Tables 1–8 are available from the “Supplementary data” link in the online posting of the article and from the same link in the online table of contents at https://academic.oup.com/jn/.

Abbreviations used:

DGA: Dietary Guidelines for Americans
FITS: Feeding Infants and Toddlers Study
NH: non-Hispanic
SSB: sugar-sweetened beverage
WIC: Special Supplemental Nutrition Program for Women, Infants, and Children
