# Supplementary material for: Room for Improvement Remains in Food Consumption Patterns of Young Children Aged 2–4 Years
Source: J Nutr. 2018 Jun 5;148(Suppl 3):1536S–1546S. doi: 10.1093/jn/nxx053 (PMC6126636; doi:10.1093/jn/nxx053)
Supplement: Supplement Tables [file nxx053_supplement_tables.docx]

**FRUIT AND 100% FRUIT JUICE**

***Baby: 100% juice***

*Apple*

*Apple blend*

*Grape*

*Orange*

*Pear*

*Punch/blend*

*Vegetable and fruit blends*

***100% juice***

*Apple*

*Apple blend*

*Berry*

*Blackberry*

*Cherry*

*Grape*

*Lemon*

*Lime*

*Mango*

*Orange*

*Orange blend*

*Peach*

*Pear*

*Pineapple*

*Pineapple blend*

*Pomegranate*

*Prune juice*

*Punch/blend*

*Strawberry*

*Vegetable and fruit blends*

*Watermelon*

***Babyfood: fruit***

*Apples/apple mixtures*

*Bananas/banana mixtures*

*Dried fruit*

*Mixed fruits*

*Other fruits/mixtures*

*Pears and pear mixtures*

***Apple/applesauce***

*Apple*

Fresh or frozen

Canned or cooked

Sweetened or syrup pack

Dried

*Applesauce*

Canned or cooked

Unsweetened or water/juice pack

Sweetened or syrup pack

Unknown pack

***Bananas***

Fresh or frozen

Dried

***Berries***

*Blackberry*

Fresh or frozen

*Blueberry*

Fresh or frozen

Dried

*Cherry*

Fresh or frozen

Dried

*Cranberry*

Fresh or frozen

Dried

*Gogi berry*

Fresh or frozen

*Raspberry*

Fresh or frozen

*Strawberry*

Fresh or frozen

Sweetened or syrup pack

Dried

***Citrus fruits***

*Grapefruit*

Fresh or frozen

*Orange*

Fresh or frozen

Canned or cooked

Unsweetened or water/juice pack

Sweetened or syrup pack

Unknown pack

*Tangelo*

Fresh or frozen

***Grapes***

*Fresh or frozen*

***Melon***

*Cantaloupe*

Fresh or frozen

*Honeydew*

Fresh or frozen

*Other melon*

Fresh or frozen

*Watermelon*

Fresh or frozen

***Mixed fruits***

*Fresh or frozen*

*Canned or cooked*

Unsweetened or water/juice pack

Sweetened or syrup pack

Unknown pack

*Dried*

***Other fruit***

*Apricot*

Fresh or frozen

Canned or cooked

Unsweetened or water/juice pack

Dried

*Avocado*

Fresh or frozen

*Dates*

Dried

*Fig*

Dried

*Kiwi*

Fresh or frozen

*Mango*

Fresh or frozen

Canned or cooked

Dried

*Nectarine*

Fresh or frozen

*Papaya*

Fresh or frozen

*Peaches*

Fresh or frozen

Canned or cooked

Unsweetened or water/juice pack

Sweetened or syrup pack

Unknown pack

*Pear*

Fresh or frozen

Canned or cooked

Unsweetened or water/juice pack

Sweetened or syrup pack

*Pineapple*

Fresh or frozen

Canned or cooked

Unsweetened or water/juice pack

Unknown pack

Dried

*Plum*

Fresh or frozen

*Pomegranate*

Fresh or frozen

*Prune*

Canned or cooked

Sweetened or syrup pack

Dried

*Raisins*

Dried

**GRAINS AND GRAIN PRODUCTS**

***Bread, rolls, biscuits, bagels, tortilla***

*Bread*

100% whole grain

Whole grain 50-99%

*Rolls*

100% whole grain

Whole grain 50-99%

*Biscuit*

*Bagel*

100% whole grain

*Tortilla*

100% whole grain

***Babyfood: cereal***

*Rice*

Dry

100% whole grain

Whole grain 50-99%

Jarred

Whole grain 50-99%

*Oat*

Dry

100% whole grain

Whole grain

Whole grain 50-99%

Jarred

100% whole grain

Whole grain 50-99%

*Quinoa*

Dry

100% whole grain

*Wheat*

Dry

100% whole grain

Whole grain 50-99%

*Multigrain*

Dry

Whole grain 50-99%

Jarred

*Unknown*

Dry

Jarred

***Cereal, family***

*Hot*

Not presweetened

100% whole grain

Not presweetened

Presweetened

Whole grain 50-99%

Presweetened

*RTE*

Not presweetened

Presweetened

100% whole grain

Not presweetened

Presweetened

Whole grain 50-99%

Not presweetened

Presweetened

***Baby: grain based finger food***

*Crackers*

Whole grain 50-99%

*Pretzels*

*Rice cake*

*Baby puffs*

*Baby cereal bars*

***Crackers, pretzels, rice cakes***

*Crackers*

100% whole grain

Whole grain 50-99%

*Pretzels*

*Rice cake*

Whole grain 50-99%

***Pancakes, waffles, and french toast***

*French toast*

100% whole grain

*Pancakes*

100% whole grain

*Waffles*

100% whole grain

***Pasta and rice***

*Pasta*

100% whole grain

*Pasta mixed dish*

*Rice*

100% whole grain

*Rice mixed dish*

***Other***

*Other grain mixed dish*

*Other grains*

100% whole grain

**MEATS AND OTHER PROTEIN SOURCES**

***Babyfood meats***

*Beef*

*Chicken or turkey*

*Pork/ham*

***Meats***

*Beef*

Breaded

*Chicken or turkey*

Breaded

*Pork*

Breaded

*Lamb, goat, game*

*Organ meats*

*Hotdogs, cold cuts, sausages, bacon*

Bacon

Cold cuts/cured meats

Hot dogs

Sausage

*Fish and shellfish*

Canned

Breaded

*Unspecified meat*

***Eggs/egg dishes***

***Dried beans, peas and legumes***

***Peanut butter, nuts, and seeds***

*Peanut butter*

***Vegetarian meat substitutes***

**MILK AND MILK PRODUCTS**

***Baby milk***

*Human milk*

*Infant formula*

Iron-fortified

*Toddler milk drink*

Iron-fortified

***Milk***

*Cow's milk*

Nonfat

Unflavored

Flavored

Powder

Lowfat

Unflavored

Flavored

Powder

Reduced fat

Unflavored

Flavored

Powder

Whole

Unflavored

Flavored

Powder

*Goat's milk*

*Plant milks/dairy substitutes*

Unflavored

Flavored

***Baby: milk products***

*Yogurt*

***Milk products***

*Cheese*

*Yogurt*

**MIXED DISHES**

***Baby food dinners***

*Beans and rice, other bean mixtures*

*Beef with vegetables and/or rice/pasta*

*Chicken or turkey with vegetables and/or rice/pasta*

*Pork or ham with vegetables and/or rice/pasta*

*Macaroni and cheese*

*Spaghetti, ravioli, lasagna*

***Beans and rice, other bean mixtures***

***Beef with vegetables and/or rice/pasta***

***Burrito, taco, enchilada, nachos***

***Chicken or turkey with vegetables and/or rice/pasta***

***Pork/ham with vegetables and/or rice/pasta***

***Fish or shellfish with vegetables and/or rice/pasta***

***Macaroni and cheese***

***Pizza***

***Soup***

***Spaghetti, ravioli, lasagna***

***Sandwich***

*Cheese*

*Hamburger or cheeseburger*

With cheese

*Hot dog*

*Meat, fish, poultry, egg*

With cheese

*Peanut butter*

**SAVORY SNACKS**

***Chips and other salty snacks***

*Chips*

100% whole grain

Whole grain 50-99%

*Corn chips*

100% whole grain

*Popcorn*

100% whole grain

*Snack mix*

*Puffs*

**SWEETS, SWEETENED BEVERAGES, AND DESSERTS**

***Babyfood desserts***

***Cereal/nutrition bars***

*100% whole grain*

*Whole grain 50-99%*

***Baby food: cookies, teething biscuits***

***Sweet bakery***

*Cakes and pies*

*Cookies, bars, brownies*

*Sweet rolls, doughnuts*

100% whole grain

*Muffins, quick breads*

***Candy***

***Ice cream, frozen yogurt, pudding***

***Gelatins, ices, sorbets***

***Sweetened beverages***

*Soft drinks*

Diet

*Fruit-flavored drink*

Diet

*Tea and coffee*

*Other*

Sports drinks

***Milk flavorings***

***Sugar, syrups, preserves, and jelly***

**VEGETABLES**

***Baby/toddler vegetables***

*Dark green vegetables*

Broccoli/broccoli mixtures

Spinach/spinach mixtures

*Orange and red vegetables*

Beets

Carrots/carrot mixtures

Squash/squash mixtures

Sweet potatoes/sweet potato mixtures

*Starchy vegetables*

Corn/corn mixtures

Peas/pea mixtures

Other

*Other vegetables*

Green beans/green bean mixtures

Mixed

***Vegetables***

*Dark green vegetables*

Broccoli

Raw

Cooked

Brussels sprouts

Cooked

Greens

Raw

Cooked

Spinach

Raw

Cooked

*Orange and red vegetables*

Beets

Raw

Cooked

Carrots

Raw

Cooked

Peppers

Raw

Cooked

Squash

Cooked

Sweet potato

Raw

Cooked

Tomatoes/tomato sauce

Raw

Cooked

*Starchy vegetables*

Corn

Cooked

Green peas

Raw

Cooked

Other

Cooked

*Other vegetables*

Asparagus

Cooked

Cabbage

Raw

Cooked

Cauliflower

Raw

Cooked

Celery

Raw

Cooked

Cucumber

Raw

Green beans

Raw

Cooked

Lettuce/green salad

Raw

Mushrooms

Cooked

Onions

Raw

Cooked

Pea pods

Raw

Cooked

Peppers

Raw

Cooked

Zucchini/summer squash

Cooked

Mixtures

Cooked

Other

Raw

Cooked

***White potatoes***

*Baked/boiled*

*Fried*

*Mashed/potato mixtures*

**OTHER**

***Water***

*Tap or bottled water*

Baby

*Flavored water*

Baby

***Fats and oils***

*Butter, margarine, animal fats*

Butter

Margarine

Cream

Other

*Salad dressings and oils*

Dressing

Mayonnaise

Oil

***Condiments and sauces***

*Condiments, herbs, and seasonings*

*Gravies and sauces*

***Supplements***

*Meal replacer*

*Other*

**Supplemental Table 2. Consumption of milk and milk products by age, all races**

|  | **Percent Consuming (%)^1^** | |
| --- | --- | --- |
| **Food** | **24 to 35.9 mo** | **36 to 47.9 mo** |
| Any liquid milk^2^ | 86±2.5 | 82±3.0 |
| Any cow’s milk^3^ | 81±2.8 | 81±3.1 |
| Whole cow’s milk^4^ | 29±3.4 | 24±3.4 |
| 2%/reduced fat cow’s milk^4^ | 29±3.5 | 30±3.4 |
| 1%/lowfat cow’s milk^4^ | 20±3.0 | 24±3.0 |
| Nonfat/skim cow’s milk^4^ | 4.2±1.3 | 3.0±0.9 |
| Plant milks/dairy substitutes^5^ | 6.2±1.6 | 2.8±1.0 |
| Any flavored milk^6^ | 15±2.6 | 16±3.3 |
| Cheese | 40±3.6 | 40±3.7 |
| Yogurt^7^ | 26±3.2 | 28±3.8 |

^1^ Values are mean percentage of children consuming the food category during a single 24-h recall ± SEs.

^2^ Includes all non-baby liquid milks, including cow’s milk, goat’s milk, and plant milks or dairy substitutes; excludes human milk (breastmilk), infant formula, and toddler milk drinks, which are not consumed among this age group.

^3^ Includes all fat levels, as well as flavored, unflavored, or powdered.

^4^ Includes only unflavored cow’s milk of specified fat level, excludes flavored and powdered.

^5^ Includes soy milk, almond milk, and other plant-based dairy substitutes; may be flavored or unflavored.

^6^ Includes flavored cow’s milk, flavored plant milks, and flavored dairy substitutes.

^7^ Excludes baby food yogurts, which are not consumed among this age group.

**Supplemental Table 3. Consumption of grains by age, all races**

|  | | **Percent Consuming (%)^1^** | |
| --- | --- | --- | --- |
| **Food** | | **24 to 35.9 mo** | **36 to 47.9 mo** |
| Any grains or grain product | 94±1.5 | | 96±1.4 |
| Any whole grain rich food^2^ | 58±3.6 | | 60±3.8 |
| Family cereal (RTE or hot)^3^ | 51±3.8 | | 54±3.9 |
| Whole-grain-rich cereal^2^ | 40±3.8 | | 42±3.8 |
| Non-whole-grain-rich cereal^2^ | 13±2.2 | | 13±2.3 |
| Presweetened cereal | 25±3.2 | | 34±3.6 |
| Not presweetened cereal | 28±3.5 | | 23±3.2 |
| Breads, rolls, biscuits, bagels, & tortillas | 47±3.7 | | 58±3.8 |
| Whole-grain-rich breads etc.^2^ | 18±2.9 | | 22±3.5 |
| Crackers, pretzels, & rice cakes | 33±3.4 | | 33±3.7 |
| Whole-grain-rich crackers etc.^2^ | 3.2±0.9 | | 2.8±1.2 |
| Pancakes, waffles, French toast | 24±3.2 | | 23±3.2 |
| Whole-grain-rich pancakes etc.^2^ | 5.9±1.9 | | 2.6±1.0 |
| Rice and pasta | 25±3.3 | | 31±4.0 |
| Rice | 13±2.7 | | 20±3.7 |
| Whole-grain-rich rice^2^ | 2.4±0.8 | | 4.6±1.5 |
| Pasta | 9.1±2.0 | | 7.1±2.0 |
| Whole-grain-rich pasta^2^ | 2.2±0.9 | | 0.8±0.4 |
| Other grain mixed dish | 1.0±0.5 | | 2.0±1.0 |

RTE: ready-to-eat

^1^ Values are mean percentage of children consuming the food category during a single 24-h recall ± SEs.

^2^ Whole-grain-rich includes products within a category that are ≥50% wholegrain. Non-whole-grain-rich includes products within a category that are <50±% wholegrains.

^3^ Includes any ready-to-eat or hot cereal; excludes infant cereals, which are not consumed among this age group.

**Supplemental Table 4. Consumption of vegetables by age, all races**

|  | **Percent Consuming (%)^1^** | |
| --- | --- | --- |
| **Food** | **24 to 35.9 mo** | **36 to 47.9 mo** |
| Any vegetable^2^ | 73±3.6 | 73±3.4 |
| Cooked vegetables^3^ | 46±3.7 | 47±3.8 |
| Raw vegetables^3^ | 24±2.9 | 17±2.4 |
| Dark-green vegetables^4^ | 17±2.7 | 14±2.8 |
| Orange and red vegetables^5^ | 27±3.2 | 20±2.8 |
| White potatoes | 29±3.3 | 35±3.8 |
| Baked/boiled | 5.1±2.1 | 2.7±1.1 |
| Mashed/other potato mixtures | 6.7±1.5 | 15±2.4 |
| Fried potatoes^6^ | 20±2.7 | 19±3.4 |
| Other starchy vegetables^7^ | 12±2.1 | 12±2.6 |
| Other vegetables^8^ | 34±3.6 | 30±3.2 |

^1^ Values are mean percentage of children consuming the food category during a single 24-h recall ± SEs.

^2^ Includes any vegetable, including white potatoes.

^3^ Excludes white potatoes; excludes baby food, which is not consumed among this age group.

^4^ Includes broccoli, Brussel sprouts, greens, and spinach; excludes baby food, which is not consumed among this age group.

^5^ Includes beets, carrots, squash, sweet potato, red peppers, tomatoes, and tomato sauce; excludes baby food, which is not consumed among this age group.

^6^ Includes French fries and any other kind of fried potatoes

^7^ Includes corn, green peas, and other starchy vegetables other than white potatoes; excludes baby food, which is not consumed among this age group.

^8^ Includes asparagus, cabbage, cauliflower, celery, cucumber, green beans, lettuce, green salad, mushrooms, onions, pea pods, peppers (not red), zucchini/summer squash, and vegetable mixtures; excludes baby food, which is not consumed among this age group.

**Supplemental Table 5. Consumption of fruit and 100% fruit juice by age, all races**

|  | | **Percent Consuming (%)^1^** | |
| --- | --- | --- | --- |
| **Food** | | **24 to 35.9 mo** | **36 to 47.9 mo** |
| Any fruit or 100% juice^2^ | 88±2.6 | | 85±2.6 |
| Any fruit^3^ | 81±3.0 | | 74±3.4 |
| Fresh or frozen fruit | 74±3.2 | | 66±3.7 |
| Canned or cooked fruit | 25±3.0 | | 21±3.1 |
| Sweetened/packed in syrup^4^ | 11±2.3 | | 11±2.7 |
| Unsweetened/packed in juice or water^4^ | 13±2.0 | | 8.7±1.7 |
| Dried fruit | 6.2±1.4 | | 4.1±1.3 |
| Any 100% fruit juice^5^ | 44±3.7 | | 45±3.9 |
| Apple/apple juice blend | 20±3.0 | | 22±3.5 |
| Grape juice | 6.5±1.8 | | 7.3±2.5 |
| Citrus/citrus juice blend | 17±2.8 | | 15±3.2 |

^1^ Values are mean percentage of children consuming the food category during a single 24-h recall ± SEs.

^2^ Includes fruit, 100±% fruit juice, and baby food fruit/100±% juice.

^3^ Includes any fruit (not juice) that is not baby food, which is not consumed among this age group.

^4^ Sweetened and unsweetened apply only to canned or cooked fruit

^5^ Includes only 100±% juices; excludes baby juice.

**Supplemental Table 6. Consumption of meats and protein foods by age, all races**

|  | **Percent Consuming (%)^1^** | |
| --- | --- | --- |
| **Food** | **24 to 35.9 mo** | **36 to 47.9 mo** |
| Any meat or other protein food^2^ | 88±2.3 | 87±2.4 |
| Any meat^3^ | 74±3.6 | 78±3.0 |
| Beef | 9.9±2.4 | 15±2.9 |
| Chicken or turkey | 38±3.6 | 43±3.9 |
| Fish or shellfish | 8.9±1.9 | 4.3±1.6 |
| Hot dogs, sausages, bacon, cold cuts | 32±3.2 | 35±3.7 |
| Pork/ham | 4.2±1.3 | 6.5±2.4 |
| Other protein sources^2^ | 47±3.8 | 49±3.9 |
| Dried beans, peas, legumes | 7.1±1.8 | 12±2.9 |
| Vegetarian meat substitutes | 1.6±1.0 | 0 |
| Eggs and egg dishes | 27±3.5 | 23±3.5 |
| Peanut butter, nuts, seeds | 20±3.0 | 25±3.7 |

^1^ Values are mean percentage of children consuming the food category during a single 24-h recall ± SEs.

^2^ Excludes cheese and yogurt, which are presented in Supplemental Table 2.

^3^ In addition to the categories listed, includes lamb, goat, game, and organ meats, which are consumed by less than 1±% of respondents in all age groups.

**Supplemental Table 7. Consumption of desserts, sugar sweetened beverages (SSBs), and sweet or savory snacks by age, all races**

|  | | **Percent Consuming (%)^1^** | |
| --- | --- | --- | --- |
| **Food** | | **24 to 35.9 mo** | **36 to 47.9 mo** |
| Any sweets, SSB, or dessert | 89±2.2 | | 91±2.1 |
| Cereal/nutrition bars | 8.3±1.6 | | 7.7±1.7 |
| Sweet bakery^2^ | 41±3.6 | | 39±3.6 |
| Ice cream, frozen yogurt, pudding | 13±2.5 | | 11±1.8 |
| Gelatins, ices, sorbets | 5.7±1.6 | | 7.7±1.8 |
| Candy | 29±3.1 | | 30±3.7 |
| Milk flavorings | 4.3±1.6 | | 5±1.6 |
| Sugar, syrup, preserves, jelly | 30±3.3 | | 41±4.0 |
| Any sweetened beverage | 41±3.7 | | 49±3.9 |
| Carbonated sodas | 6.9±1.7 | | 11±2.5 |
| Fruit flavored drinks | 32±3.5 | | 37±3.6 |
| Sweetened tea and coffee | 8.1±2.1 | | 5.7±1.6 |
| Other | 2.4±0.9 | | 5.6±2.2 |
| Sports drinks | 1.0±0.6 | | 2.6±1.3 |
| Any savory snacks^3^ | 37±3.6 | | 35±3.7 |
| Whole-grain-rich savory snacks^4^ | 21±3.3 | | 14±2.2 |

^1^ Values are mean percentage of children consuming the food category during a single 24-h recall ± SEs.

^2^ Includes cakes, pies, chocolate/sweet cookies, bars, brownies, sweet rolls, doughnuts, muffins, and quick breads.

^3^ Includes chips, corn chips, popcorn, snack mix, and puffs (non-babyfood), regardless of wholegrain content.

^4^ Includes savory snacks that are ≥50% whole grain.

**Supplemental Table 8. Consumption of mixed dishes by age, all races**

|  | **Percent Consuming (%)^1^** | |
| --- | --- | --- |
| **Food** | **24 to 35.9 mo** | **36 to 47.9 mo** |
| Any mixed dishes^2^ | 71±3.1 | 63±3.8 |
| Beans and rice, other bean mixtures | 6.9±2.6 | 2.8±2.1 |
| Beef with vegetables and/or rice/pasta | 7.6±2.7 | 2.5±1.1 |
| Burrito, taco, enchilada | 8.8±2.5 | 5.4±1.6 |
| Chicken or turkey with vegetables and/or rice/pasta | 2.8±1.0 | 2.4±1.1 |
| Pork/ham with vegetables and/or rice/pasta | 0.8±0.7 | 0 |
| Fish or shellfish with vegetables and/or rice/pasta | 2.6±2.2 | 0.8±0.5 |
| Macaroni and cheese | 14±2.4 | 11±2.0 |
| Spaghetti, ravioli, lasagna | 11±2.3 | 12±2.4 |
| Pizza | 13±2.6 | 15±2.6 |
| Sandwich | 15±2.6 | 21±3.3 |
| Soup | 16±3.7 | 11±2.6 |

^1^ Values are mean percentage of children consuming the food category during a single 24-h recall ± SEs.

^2^ Excludes mixed dishes that are predominantly grains, which are presented in Supplemental Table 3.
